# Comparative proteomic analyses demonstrate enhanced interferon and STAT-1 activation in reovirus T3D-infected HeLa cells

**DOI:** 10.3389/fcimb.2015.00030

**Published:** 2015-04-07

**Authors:** Peyman Ezzati, Krysten Komher, Giulia Severini, Kevin M. Coombs

**Affiliations:** ^1^Manitoba Centre for Proteomics and Systems Biology, University of ManitobaWinnipeg, MB, Canada; ^2^Department of Medical Microbiology, Faculty of Medicine, University of ManitobaWinnipeg, MB, Canada; ^3^Manitoba Institute of Child Health, John Buhler Research CentreWinnipeg, MB, Canada

**Keywords:** RNA virus, virus infection, host cell alterations, mass spectrometry, liquid chromatography, cell signaling, bioinformatics

## Abstract

As obligate intracellular parasites, viruses are exclusively and intimately dependent upon their host cells for replication. During replication viruses induce profound changes within cells, including: induction of signaling pathways, morphological changes, and cell death. Many such cellular perturbations have been analyzed at the transcriptomic level by gene arrays and recent efforts have begun to analyze cellular proteomic responses. We recently described comparative stable isotopic (SILAC) analyses of reovirus, strain type 3 Dearing (T3D)-infected HeLa cells. For the present study we employed the complementary labeling strategy of iTRAQ (isobaric tags for relative and absolute quantitation) to examine HeLa cell changes induced by T3D, another reovirus strain, type 1 Lang, and UV-inactivated T3D (UV-T3D). Triplicate replicates of cytosolic and nuclear fractions identified a total of 2375 proteins, of which 50, 57, and 46 were significantly up-regulated, and 37, 26, and 44 were significantly down-regulated by T1L, T3D, and UV-T3D, respectively. Several pathways, most notably the Interferon signaling pathway and the EIF2 and ILK signaling pathways, were induced by virus infection. Western blots confirmed that cells were more strongly activated by live T3D as demonstrated by elevated levels of key proteins like STAT-1, ISG-15, IFIT-1, IFIT-3, and Mx1. This study expands our understanding of reovirus-induced host responses.

## Introduction

The mammalian orthoreoviruses (MRV) are non-enveloped viruses that contain a genome comprising 10 segments of double-stranded (ds)RNA. The dsRNA genome is enclosed in a concentric double-layered protein capsid built from eight different viral structural proteins. For reviews, see Danthi et al. (2010), Coombs (2011b), and Dermody et al. (2013). MRV are the prototype members of the family *Reoviridae*, genus Orthoreovirus. The Ortheoreoviruses include fusogenic avian reovirus and non-fusogenic MRV and the *Reoviridae* family also contains rotaviruses (Estes and Kapikian, 2007), orbiviruses (Roy, 2007), and at least 10 other genera, divided into two sub-families based upon particle morphology (Mertens et al., 2005; Coombs, 2011b; Dermody et al., 2013). MRV infections are generally mild in humans but most other family members are highly pathogenic in their respective hosts. MRV currently consist of three generally studied serotypes, with each represented by a prototype strain: strain Lang (T1L) for serotype 1; strain Jones (T2J) for serotype 2, and strain Dearing (T3D) for serotype 3. A possible fourth strain, Ndelle virus, has also been proposed (Attoui et al., 2001). MRV have long served as models for understanding viral pathogenesis (Dermody et al., 2013) and they may also be oncolytic agents (Coffey et al., 1998; Forsyth et al., 2008; Thirukkumaran et al., 2010) because of their capacity to selectively kill cancer cells that contain functional p53 and an activated Ras pathway (Coffey et al., 1998; Pan et al., 2011).

Virus infection induces numerous alterations in cells. Many such alterations have been detected and measured at the mRNA level by gene array analyses (see for example, Geiss et al., 2002; Poggioli et al., 2002; Debiasi et al., 2003; Kobasa et al., 2007; Tyler et al., 2010). However, since mRNA levels cannot provide complete information about types of post-translational modifications or levels of protein synthesis, the utility of such studies for predicting cellular proteomic responses is usually limited (Pradet-Balade et al., 2001; Tian et al., 2004; Baas et al., 2006). Therefore, quantitative and comparative proteomic analyses have been used to provide complementary information about host responses to virus infection (reviewed in Yates et al., 2009; Coombs, 2011a). Commonly used methods include 2-dimensional difference in gel electrophoresis (2D-DIGE (see for examples, Burgener et al., 2008; Lucitt et al., 2008), and newer non-gel-based strategies such as stable isotope labeling by amino acids in cell culture (SILAC, Ong et al., 2002; de Hoog et al., 2004; Everley et al., 2004; Yan et al., 2004; Ong and Mann, 2005), isotope coded affinity tags (ICAT, Booy et al., 2005; Stewart et al., 2006), and isobaric tags for relative and absolute quantitation (iTRAQ, Dwivedi et al., 2009; Zhang et al., 2009). Li and colleagues used 2D-DIGE of MRV-infected murine myocytes and found regulation of several proteins, including heat shock proteins and interferon-response proteins (Li et al., 2010). We previously used SILAC to label reovirus T1L-infected HEK293 cells (Berard et al., 2012) and T3D-infected HeLa cells (Jiang et al., 2012; Coombs, 2013) with light and heavy isotopic arginine and lysine to compare these infected cells to reciprocally-labeled mock-infected cells. The non-gel-based approaches generally identify more proteins than the gel-based approaches and also are usually better at measuring down-regulated proteins (Yates et al., 2009; Coombs, 2011a). SILAC is a simple and straightforward method but is usually limited to analyzing and comparing a limited number of samples. By contrast, iTRAQ (Choe et al., 2005; Prange and Proefrock, 2008) allows simultaneous analysis of four or more samples.

The above SILAC analyses successfully identified and measured several thousand host proteins, many of which are involved in cell death, cell growth and proliferation, molecular transport, gene expression, and inflammatory response pathways (Berard et al., 2012; Jiang et al., 2012; Coombs, 2013) but the cellular proteomic repertoire is several orders of magnitude larger. In addition, the non-gel-based strategies, which generally successfully identify significantly more proteins than the gel-based 2D-DIGE strategies (Yates et al., 2009; Coombs, 2011a), were used to analyze single reovirus strains. Thus, as part of an ongoing systematic effort to delineate reovirus-induced host protein responses, we extended our proteomic analyses by examining multiple reovirus clones and by using a complementary approach. Although there are 3–4 reovirus serotypes, the most extensively studied are T1L and T3D. In addition, cellular perturbations, including signaling, may be caused by active viral replication, induced by live virus, or by engagement of non-infectious virus with intracellular and extracellular components. Thus, we chose to take advantage of the multi-plexing capacity of iTRAQ by analyzing live T1L- and T3D-infected cells, and comparing responses to mock and dead T3D, a total of four conditions. We identified and measured 2375 proteins with two or more peptides at >99% confidence and found that 137 were significantly quantitatively regulated. There also were major differences in the proteins and pathways induced by T1L, T3D, and UV-T3D. For example, T3D induced significant up-regulation of several proteins in the Interferon signaling pathway, including STAT-1, IFIT-1, IFIT-3, and Mx1.

## Materials and methods

### Cells and viruses

#### Cell lines and media

Mouse L929 fibroblast cells (L929) were grown in Joklik's suspension minimal essential medium (J-MEM) (Gibco, Grand Island, NY) supplemented to contain 5% fetal bovine serum (FBS) (Invitrogen Canada Inc., Burlington, Ontario), and 2 mM L-glutamine as described (Berard and Coombs, 2009). Cells were sub-cultured daily. Human HeLa cells were cultured as monolayers in Dulbecco's modified MEM (D-MEM) supplemented with 0.2% (w/v) glucose, 10% FBS (Invitrogen), 2 mM l-glutamine, non-essential amino acids, and sodium pyruvate as described (Coombs, 2013). Cells were sub-cultured 2–3 times each week.

#### Viruses

Reovirus strain type 1 Lang (T1L) and type 3 Dearing-Fields (T3D) are laboratory stocks. Virus stocks were usually grown in L929 cell monolayers in J-MEM in the presence of 5% CO_2_ at 37°C as above, but with 3% FBS, 100 U/ml of penicillin, 100 μg/ml streptomycin sulfate, and 100 μg/ml amphotericin-B as previously described (Berard and Coombs, 2009). Virus titers were determined on L929 monolayers as described (Berard and Coombs, 2009).

#### Virus purification

Large quantities of reovirus T1L and T3D were grown in suspension L929 cell cultures and purified by routine Vertrel-XF™ extraction and cesium chloride ultracentrifugation procedures (Mendez et al., 2000). Gradient-purified virions were harvested, dialyzed against 2× SSC Buffer (150 mM NaCl, 15 mM Na-citrate, pH 7.0) and virus concentrations were measured spectrophotometrically, using the relationship 1 ODU_260_ = 2.1 × 10^12^ particles/ml (Smith et al., 1969). Aliquots of purified T3D were inactivated by exposure to an ultra-violet light box. Non-treated T1L and T3D infectivity, and UV-induced T3D loss of infectivity, were confirmed by plaque assay (Berard and Coombs, 2009).

### Infections

For non-iTRAQ analyses (i.e., for Western blot validation—see below), cells were mock-treated or were infected with T1L, T3D, or UV-T3D, harvested at specified time points, and fractionated as described below. For iTRAQ labeling, sets of HeLa cells were infected with gradient-purified T1L or T3D at a multiplicity of infection (MOI) of seven PFU per cell, or with an equal number of UV-inactivated T3D (UV-T3D) particles, or were mock-infected with diluent. Experiments were performed three times.

### Cell fractionation

Infected and mock-infected cells were harvested at various times post-infection. Harvested cells were washed three times in >50 volumes of ice-cold Phosphate Buffered Saline (PBS). Three times-washed cells were resuspended in ice-cold PBS supplemented with 1.5 times Complete™ Protease Inhibitor (Pierce) at a concentration of ~10^7^ cells per 0.3 ml and cells were lysed by the addition of one-tenth volume of 5% NP-40. Cells were incubated for 30 min with periodic mixing and nuclei pelleted at 500 × g for 10 min. Supernatants (cytosol and soluble membranes) were transferred to fresh tubes and the nuclei were washed four times with PBS + 0.25 times Complete™ Protease Inhibitor + 10% sucrose. Washed nuclei were extracted by a two-step MS-compatible high salt/urea procedure (Kroeker et al., 2012). Each fraction was desalted using HiTrap desalting column connected to an AKTA FPLC (GE). Protein content in every cytosolic and nuclear fraction was determined by BCA Protein Assay (Pierce) using bovine serum albumin standards. The cytosolic and nuclear fractions were stored at −80°C until further processing took place.

### Immunoblotting

Equivalent cytosolic and nuclear fractions were resolved in 10% linear mini sodium dodecyl sulfate polyacrylamide gels (SDS-PAGE, 8.0 × 6.5 × 0.1 cm) at 180 V for 60 min. Proteins were transferred to 0.2 μm polyvinylidene difluoride (PVDF) membranes at 20 V for 40 min with a Semi-dry apparatus (BioRad), and protein transfer was confirmed by Ponceau-S staining. The membranes were blocked with 5% (w/v) skim milk in Tris-buffered saline with Tween-20 (TBST; 50 mM Tris, 150 mM NaCl, 0.05% Tween 20, pH 7.4) and probed with various primary antibodies. Primary antibodies were: in-house produced rabbit anti-reovirus, rabbit α-GAPDH (Cell Signaling, cat#2118), α-ISG15 (Rockland, cat#200-401-438), and α-IFIT (Abcam, cat#ab55837); goat α-Mx1 (Santa Cruz #sc-34128); and mouse α-Actin (Sigma, cat#A5441), and α-STAT1 (Cell Signaling, cat#9176). Appropriate secondary horseradish peroxidase (HRP)-conjugated horse anti-mouse or goat anti-rabbit (Cell Signaling, cat#7076, cat#7074, respectively), or rabbit anti-goat (Zymed, cat#81-1620) were used to detect immune complexes. Bands were developed by enhanced chemiluminescence and imaged with an Alpha Innotech FluorChemQ MultiImage III instrument.

### Comparative iTRAQ analyses

#### Protein digestion and peptide fractionation

Fractionated samples were labeled with iTRAQ according to the manufacturer's instructions (Applied Biosystems, Foster City, CA, USA) and by routine procedure as previously described (Dwivedi et al., 2009; Summers et al., 2012). Briefly, for each sample, 100 μg of protein was mixed with 100 mM ammonium bicarbonate, reduced with 10 mM dithiothreitol, and incubated at 57°C for 30 min. Proteins were then alkylated with 50 mM iodoacetamide in the dark at room temperature for 30 min. Excess iodoacetamide was quenched with 17 mM dithiothreitol. Peptides were digested with one-fiftieth trypsin (w/w; Promega, Madison, WI) at 37°C for 10 h. Samples were then acidified with an equal volume of 3% trifluoroacetic acid (TFA), lyophilized, and re-suspended in 200 μL of 0.1% TFA. Samples were loaded on a C18 X-Terra column (1 × 100 mm, 5 μm, 100 Å; Waters Corporation, Milford, MA, USA), desalted using 0.1% TFA, and peptides eluted with 50% acetonitrile. Desalted samples were stored at −80°C for 2D-HPLC-MS/MS analysis. For comparative proteomic analysis, each peptide sample (100 μg) was labeled with isobaric Tags for Relative and Absolute Quantitation (iTRAQ) reagent (Applied Biosystems, Foster City, CA, USA) as outlined by the manufacturer. For two experimental replicates, the cytosolic or nuclear fractions were labeled with either the “even”-numbered probes (MW = 114, 116, 118, 121) of an iTRAQ 8-plex reagent kit or with the odd-numbered probes (MW = 113, 115, 117, 119). For the third experimental replicate, the cytosolic and nuclear fractions were divided in half and each fraction was labeled with all eight iTRAQ probes, providing four sets of measurements for the three biological replicates.

#### Two-dimensional high-performance liquid chromatography-mass spectrometry

Labeled peptides were mixed in equal proportions and subjected to 2D-HPLC-MS/MS (Aggarwal et al., 2006; Zieske, 2006). Trypsinized peptides were separated in the first dimension with an Agilent 1100 Series HPLC system (Agilent Technologies, Wilmington, DE). Samples were injected onto a C18 X-Terra column (1 × 100 mm, 5 μm, 100 Å; Waters Corporation, Milford, MA, USA) and eluted with a linear water-acetonitrile gradient (20 mM ammonium formate, pH 10, in both eluents A and B, 1% acetonitrile/min, 150 μL/min flow rate). A concentrated 200 mM solution of ammonium formate at pH 10 was prepared as described by Gilar et al. (2005). For first-dimension separation, Buffers A and B were prepared by a one-tenth dilution of this concentrated buffer with water and acetonitrile, respectively. Fifty 1-min cytoplasmic fractions were collected (≈6.6 μg/fraction). Samples were concatenated (fraction 1 and 26, 2 and 27, etc.) into a total of 25 fractions as described by Dwivedi et al. (2008) and each concatenated fraction was lyophilized and re-suspended in 100 μL of 0.1% formic acid. Because of lower protein content, nuclear samples were collected as 30 1-min fractions and concatenated into 15 combined fractions. Protein content in each concatenated sample was determined by Nanodrop® and 2 μg of each peptide fraction was separated in the second dimension on a splitless nanoflow Tempo LC system (Eksigent, Dublin, CA, USA) with 20 μL sample injection via a 300 μm × 5 mm PepMap100 precolumn and a 100 μm × 150 mm analytical column packed with 5 μm Luna C18(2) (Phenomenex, Torrance, CA). Both eluents A (2% acetonitrile in water) and B (98% acetonitrile) contained 0.1% formic acid as ion pairing modifier. A 0.33% acetonitrile/min linear gradient (0–30% B) was used for peptide elution, providing a total 2 h run time per fraction in the second dimension.

#### Mass spectrometry

A QStar Elite mass spectrometer (Applied Biosystems, Foster City, CA) was used in standard MS/MS data dependent acquisition mode with a nano-electrospray ionization source operated in positive ion mode. One-second survey MS spectra were collected (m/z 400–1500) followed by three MS/MS measurements on the most intense parent ions (80 counts/s threshold, +2 to +4 charge state, m/z 100–1500 mass range for MS/MS), using the manufacturer's iTRAQ and “smart exit” settings. Previously targeted parent ions were excluded from repetitive MS/MS acquisition for 60 s (50 mDa mass tolerance). Standard QTOF search settings were used: 100 ppm and 0.4 Da mass tolerance for parent and fragment ions, respectively.

#### Database search, protein identification, and statistical analysis

***Raw spectra***

WIFF files containing MS and MS/MS data were analyzed using Protein Pilot 4.0 software using Paragon algorithm (Applied Biosystems). Protein identification and quantification search parameters were as follows: iTRAQ 4-plex (peptide labeled) or iTRAQ 8-plex (peptide labeled), carbamidomethylation of cysteine. Information for all 25 fractions of each sample were searched against human gene database (NCBI released March 2011, downloaded from ftp://ftp.ncbi.nih.govrefseqH_sapiensmRNA_Prot, containing 37,391 entries). Proteins, their confidences, and their expression ratios, expressed as infected: mock (I:M), were returned with gi accession numbers. Proteins identified at >99% confidence (Unused Score >2.00) for which peptides were detected at >95% confidence were used for subsequent comparative quantitative analysis. The false discovery rate (FDR), defined as the percentage of reverse proteins identified against the total protein identification, was determined to be <0.8%.

Each of the 24 datasets were normalized, essentially as described (Keshamouni et al., 2009; Coombs et al., 2010) to allow dataset merging and comparison. Briefly, every infected-to-mock (I:M) ratio was converted into log_2_ space, geometric means and standard deviations of each dataset were calculated, and every protein's log_2_ I:M ratio was converted into a z-score, using the formula:

$$\begin{array}{l} \text{z-score }(\sigma )\text{ of }[b]\\ \;=\frac{{\text{Log}}_{\text{2}}\text{I }:\text{ M}[\text{b}]\;-\text{ Average of }(\log_{2}\text{ of each member},\text{ a}\ldots .\text{n})}{\text{Standard deviation of }(\log_{2}\text{ of each member},\text{ a}\ldots .\text{n})} \end{array}$$

where “*b*” represents an individual protein in the dataset *a*….*n*, and z-score represents the number of standard deviation units (expressed as “σ”) that protein's log_2_ I:M ratio is from its population mean. Thus, a protein with a z-score >1.960 σ indicates that protein's differential expression lies outside the 95% confidence level, >2.576 σ indicates outside the 99% confidence level, and 3.291 σ indicates 99.9% confidence. z-Scores >1.960 were considered significant. Gi numbers of all significantly regulated proteins were converted into HGNC identifiers by Uniprot (http://www.uniprot.org/) and HGNC terms were submitted to and analyzed by STRING (Von Mering et al., 2007; Szklarczyk et al., 2011) and by the DAVID bioinformatic suite at the NIAID, version 6.7 (Dennis et al., 2003; Huang et al., 2009) and gene ontologies examined with the “FAT” datasets. The gi numbers were also submitted to, and pathways constructed with, Ingenuity Pathway Analysis software (IPA™).

## Results

### T1L, T3D, and UV-T3D induce different host protein regulation

**Figure 1:**
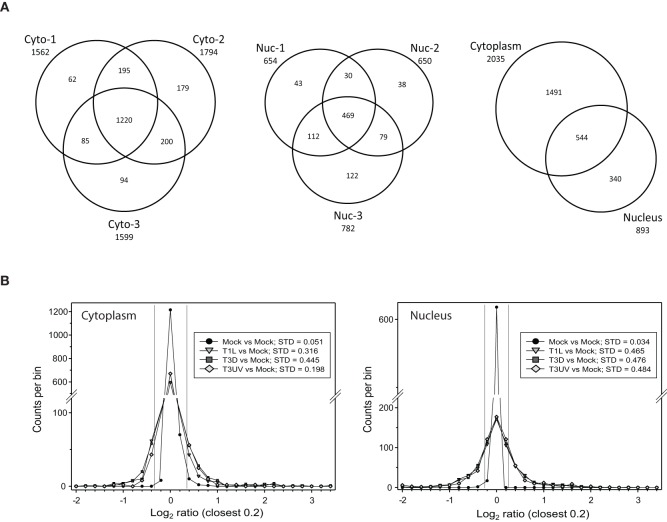
**Distributions of proteins identified in various experiments. (A)** Venn diagrams of numbers of identified proteins from various experimental replicates and cell fractions. Cells that were mock infected, or infected with reovirus T1L or T3D, or treated with UV-inactivated T3D, were harvested at 24 hpi, fractionated into the cytosolic (Cyto, left) and nuclei (Nuc, center) fractions and labeled with iTRAQ reagents. Numbers of proteins identified with two or more peptides and at >99% confidence are indicated for each of the three experimental replicates. The total numbers of unique proteins identified in the cytosolic and nuclear fractions are indicated at right. **(B)** Frequency distributions of identified proteins and their expression ratios (expressed as Log_2_ fold change compared to Mock) in representative 8-plex iTRAQ samples. The two mock samples were compared to each other and to each of the other six treatments. Standard deviations of the variance in each cytoplasmic (left) and nuclear (right) sample are shown in the box in each plot. The two thin vertical lines represent ±7 σ, corresponding to the limits within which virtually all mock samples fall. Positive values represent up-regulated host proteins in virus-infected cells; negative values represent down-regulated host proteins. Only the distributions of one set of each treatment are shown for clarity.

Titration of the gradient-purified virions and comparisons to optically-determined particle counts indicated that the particle-to-PFU ratios of the various virus samples were: T1L: 191; T3D: 327; and UV-treated T3D: >3.5 × 10^8^, respectively. These different virus preparations were then used to infect HeLa cells, cells were harvested at 24 hpi, and cytosolic and nuclear fractions prepared and analyzed as described in Materials and Methods. After removal of proteins identified at <99% confidence and those identified by a single peptide, a total of 1562, 1794, and 1599 proteins were detected and relative quantities measured in the two 4-plex and one 8-plex cytosolic fractions, respectively, and 654, 650, and 782 proteins were measured in the corresponding nuclear fractions (Figure 1A, left and center). 1491 proteins were found exclusively in the cytosol, 340 were found just in the nucleus, and 544 were found in both fractions (Figure 1A, right). Experimental variability was also assessed by examination of the 8-plex technical replicates. The two mock samples were compared to each other and to the various infections (Figure 1B). The standard deviations of Infected:Mock Log_2_ ratios were 0.051 and 0.034 for the cytosolic and nuclear Mock replicates, respectively, and 3.9- to 14-fold higher for each of the infected samples, indicating that host protein ratios were greatly affected by the virus treatments.

Each dataset was subjected to Z-score analysis to identify proteins that were significantly regulated. This analysis indicated that fold changes of ±1.29 to ±1.56 (depending upon specific dataset) could be considered significant. Thus, for further consideration, protein regulation needed to exceed ±1.29 and have a Z-score >1.960 or <-1.960. A total of 137 proteins appeared to be regulated by one or more of the virus preparations (Table 1). Twenty nine proteins were significantly up-regulated in the cytosolic fractions by T1L and 18 nuclear proteins were also up-regulated by T1L. BAF, the barrier to autointegration factor 1, was detected and found to be up-regulated in both fractions by T1L infection. Sixteen cytosolic and 14 nuclear proteins were found to be down-regulated, with no down-regulated proteins appearing in both fractions. Similarly, T3D infection resulted in determination of 32 up-regulated proteins in the cytosol and 19 up-regulated in the nucleus, with caltractin up-regulated in both fractions, and 10 and 11 down-regulated proteins in the cytosolic and nuclear fractions, respectively. UV-inactivated T3D treatment of cells resulted in 22 up-regulated proteins in the cytoplasm, 17 up-regulated in the nucleus, 19 down-regulated in the cytoplasm, and 17 down-regulated in the nucleus.

**Table 1:**
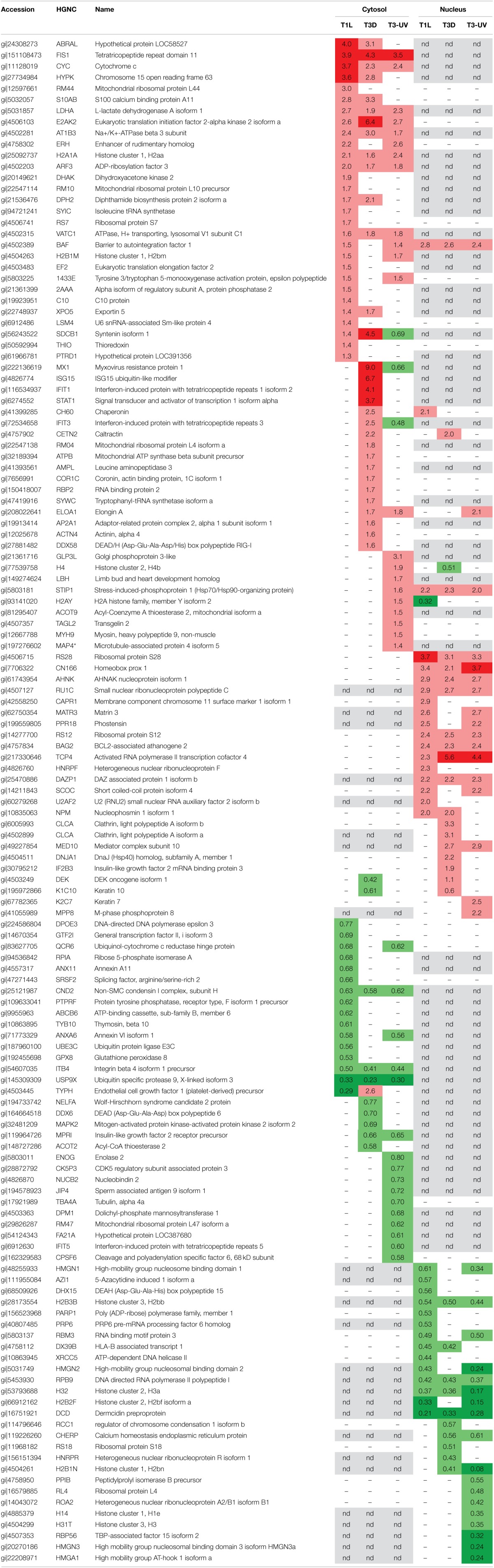
**Significantly-regulated HeLa cell proteins**.

Red and pink indicate degrees of up-regulation; green indicates down-regulation; –, not significantly regulated; n.d., not detected; ^*^Record removed from NCBI.

Several proteins were similarly regulated by all three virus treatments. Cytosolic FIS1, cytochrome c, LDHA, E2AK2, AT1B3, H2A1A, ARF3, VATC1, and nuclear STIP1, RS28, CN166, AHNK, RU1C, RS12, BAG2, TCP4, and DAZP1 were up-regulated by both live T1L and T3D and by inactivated T3D (Table 1). Cytosolic CND2, ITB4, USP9X, and nuclear H2B3B, RPB9, H32, and DCD were down-regulated by all three virus types. Many proteins were differentially regulated by only some of the three virus types. Nuclear syntenin isoform 1 (SDCB1) was not detected, but cytosolic SDCB1 was strongly up-regulated by T3D, weakly up-regulated by T1L, and down-regulated by UV-T3D. Several proteins were differentially regulated by one virus serotype but not by the other virus serotype. For example, T1L induced up-regulation of more than a dozen proteins (including RM44, ERH, DHAK, BAF, and EF2) but T3D did not induce significant alterations in levels of these proteins. Conversely, T3D induced strong up-regulation of four cytosolic immune-regulated proteins (Mx1, ISG15, IFIT1, and STAT-1) and up-regulation of more than a dozen other proteins (including IFIT3, CETN2, RBP2, and DDX58), but T1L induced non-significant changes in these proteins' levels.

### Validation of protein ratios by western blotting

**Figure 2:**
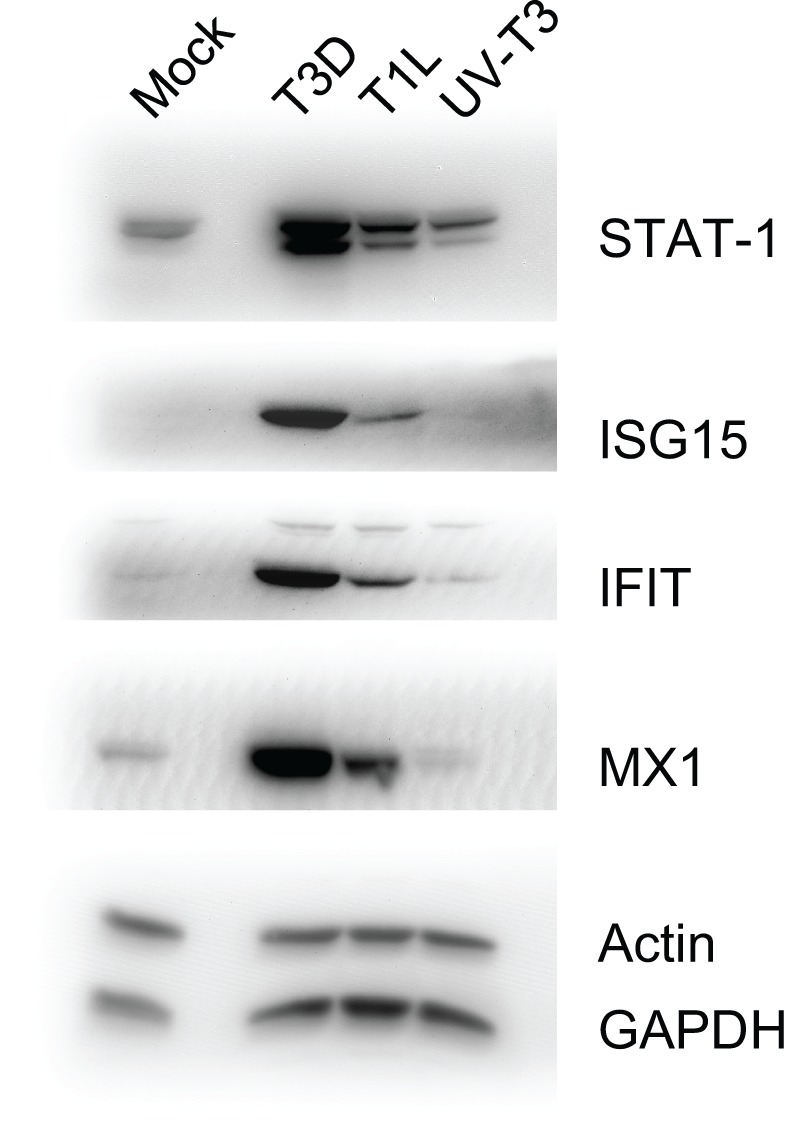
**Immunoblot analyses of indicated host proteins in HeLa cells mock-infected, or infected with T3D or T1L, or treated with UV-inactivated T3D**. Cells were harvested and lysed with 0.5% NP-40 detergent, nuclei removed, and cytosolic fractions dissolved in SDS electrophoresis sample buffer, resolved in 10% mini SDS-PAGE, transferred to PVDF, and probed with various antibodies. Bands were visualized, and intensities measured, with an Alpha Innotech FluorChem®Q MultiImage® III instrument.

To confirm some of the iTRAQ-determined protein ratios, we analyzed selected proteins in T1L- and T3D-infected, and UV-T3D-treated, cells. Immunoblotting confirmed that STAT-1, ISG15, IFIT1, and Mx1 were strongly up-regulated in T3D-infected cells but only weakly up-regulated in corresponding T1L-infected and UV-T3D-treated cells (Figure 2).

### Cell death and survival, cell signaling, infectious diseases and interferon-induced pathways are differentially induced by T1L, T3D, and UV-T3D

**Figure 3:**
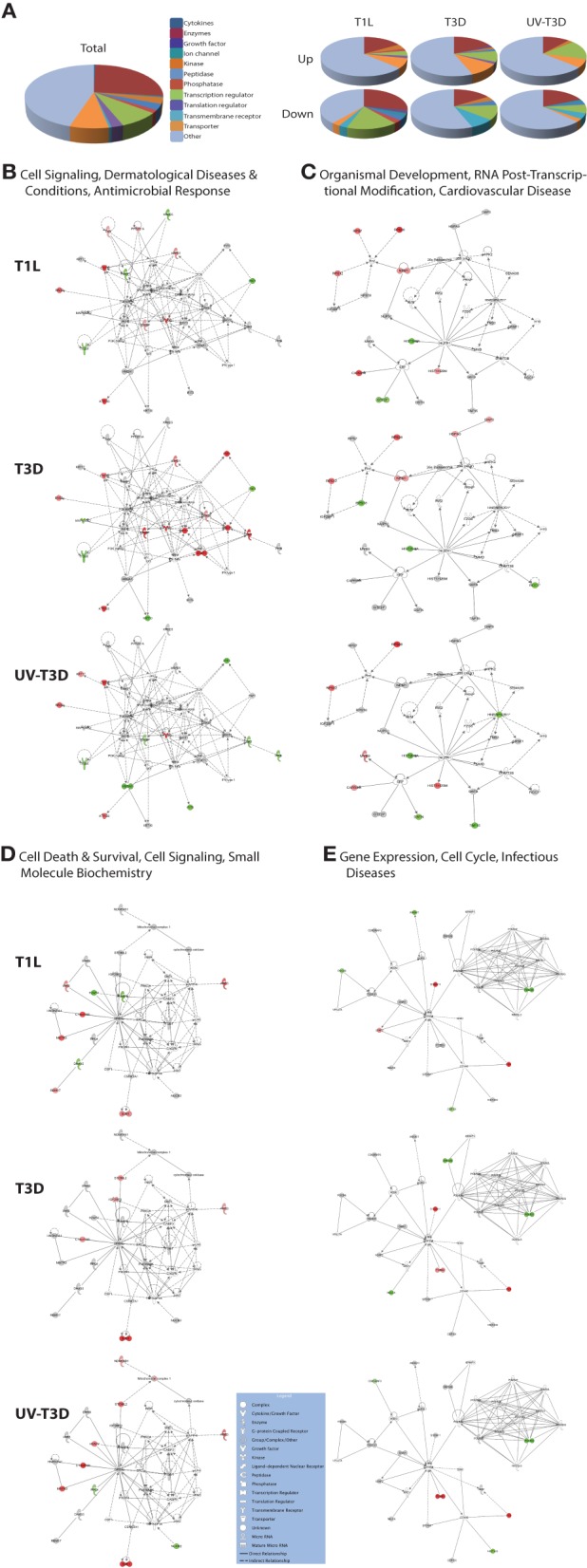
**Molecular pathways of regulated proteins**. Proteins and their levels of regulation were imported into the Ingenuity Pathways Analysis (IPA®) tool and interacting pathways were constructed. **(A)** Ontological classifications of all measured proteins (Total) as well as those significantly up- and down-regulated by each of the viruses. (**B–E)** The top four IPA networks, identified at 95% confidence and each of which contained 12 or more “focus” molecules (molecules significantly up- or down-regulated), with pathway names indicated. Solid lines: direct known interactions; dashed lines: suspected or indirect interactions. Significantly regulated proteins identified in either the cytosolic or nuclear fractions were overlaid onto each network; red, significantly up-regulated proteins; pink, moderately up-regulated proteins; gray, proteins identified but not significantly regulated; light green, moderately down-regulated proteins; dark green, significantly down-regulated proteins; white, proteins known to be in network, but not identified in our study. Molecular classes are indicated in legend. Additional networks, also with 12 or more “focus” molecules, are depicted in Supplementary Figure S1.

**Figure 4:**
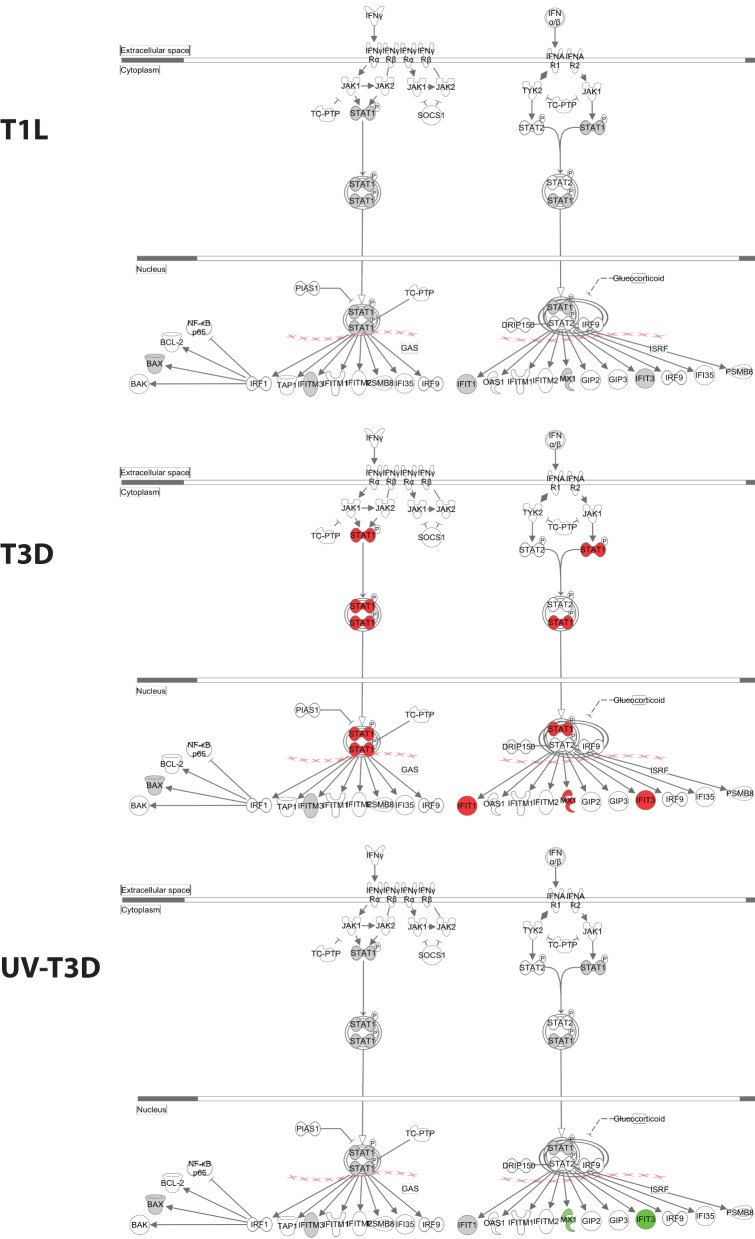
**Significantly affected canonical pathway “interferon signaling”, as determined by IPA® analysis**. Red indicates highly up-regulated proteins, light green indicates down-regulated, gray represents not significantly affected, and white represents molecules known to be part of the pathway but not identified by iTRAQ. Examples of additional affected canonical pathways are depicted in Supplementary Figure S2.

The differentially-regulated proteins were analyzed by a variety of software tools. Ingenuity Pathway Analyses (IPA) globally mapped all genes into cytokines, enzymes, growth factors, and other categories (Figure 3A). There were significant differences in the proportions of enzymes, transcription regulators, transporters, and other GO classes differentially regulated by the three virus treatments (Figure 3A). Mapping up- and down-regulated proteins into IPA networks identified nine networks that contained at least 12 focus molecules. The four highest scoring networks were: cell signaling, dermatological diseases and conditions, antimicrobial response (Figure 3B); Organismal development, RNA post-transcriptional modification, cardiovascular disease (Figure 3C); Cell death and survival, cell signaling, small molecule biochemistry (Figure 3D); and Gene expression, cell cycle, infectious diseases (Figure 3E). Additional major networks included: Cancer, Immunological disease; Inflammatory disease and response; Tissue development; DNA replication; and Cell cycle and cellular development (Supplementary Figure S1). All networks showed significant differences in the specific members that were up-regulated, non-regulated, or down-regulated by T1L, T3D, or UV-T3D. Similarly, IPA analysis identified numerous significantly-affected canonical pathways. The Interferon signaling pathway was differentially affected by the three different virus treatments (Figure 4). As reflected by differences in respective protein levels (Table 1), numerous members of this pathway were up-regulated by T3D infection but not by T1L infection, and Mx1 and IFIT3 were down-regulated by UV-T3D treatment. Representative additional canonical pathways, such as Activation of IRF by cytosolic pattern recognition receptors, EIF2 signaling, ILK signaling, and Mitochondrial dysfunction, were also differentially regulated by the three different virus treatments (Supplementary Figure S2).

## Discussion

Recent work by us and by others has begun to detect and measure quantitative differences in host mRNA and proteins induced by MRV infection. Poggioli et al. (2002) identified numerous cell cycle protein genes whose transcription was affected by either T1L or type 3 Abney infection. O'donnell et al. (2006) identified >100 interferon- and NF-κB-responsive genes that were affected by T3D infection. Transcriptomic analyses of MRV T3D- and flavivirus-infected murine brain tissues indicated up-regulation of apoptosis, interferon, inflammation and cell death/survival signaling and down-regulation of glutamate signaling genes (Clarke et al., 2014). Global protein analyses, using 2D-DIGE/MS analyses of murine cardiac myocytes infected with T1L, T3D, potently myocarditic reassortant 8B, and a non-myocarditic derivative virus DB93A, identified several thousand proteins and found 67 host proteins were differentially expressed (*p* < 0.05) (Li et al., 2010). These proteins included Hsp25 and proteins associated with amino acid metabolism, calcium signaling, ERK/ MAPK signaling, mitochondrial dysfunction, oxidative stress, and protein ubiquitination. Myocarditic reassortant virus 8B was also uniquely associated with regulation of proteins involved in endoplasmic reticulum stress and phospholipid degradation. Non-gel-based SILAC analyses of individual MRV clones identified 104 differentially expressed host proteins in T1L-infected HEK-293 cells at 6 or 24 hpi (Berard et al., 2012), and 133 (Jiang et al., 2012) and 89 (Coombs, 2013) differentially expressed proteins in T3D-infected HeLa cells. We also very recently analyzed host protein alterations in HEK-293 cells infected with each of two different T3D clones (Berard et al., accepted). Up-regulated proteins in these studies were associated with antimicrobial and antiviral responses, cell death and growth factors, GTPase activity, nucleotide binding, oxygen transport, interferon signaling, and enzymes associated with energy generation. Down-regulated proteins included those involved in apoptosis, cell and biological adhesion, isomerase activity, macromolecular catabolic activity, regulation of cell proliferation, structural molecule activity, and numerous molecular binding activities. Not all processes were affected by all virus types or in different cell types. Thus, the current study was undertaken to directly compare two virus serotypes, and live vs. dead T3D, in a common cell type that had been previously used. Comparisons of the results obtained in the current study with a previous SILAC assay of the same virus/cell system (Coombs, 2013) indicated that 1711 common proteins were identified and measured in both studies. There was good correlation between the two studies. Seven proteins (Mx1, ISG15, IFIT1, IFIT3, STAT1, E2AK2, and TYPH) were determined as up-regulated by both assays. 1660 proteins were indicated as not significantly regulated in both assays and no proteins were indicated as significantly up-regulated in one assay but down-regulated in the other.

As has been observed in earlier studies, T1L and T3D differentially induce host protein responses. This has been observed both in the single previous global proteomic screen (Li et al., 2010), and in more targeted analyses that focused on individual molecules or small sets of molecules, including a study by Tyler and colleagues demonstrating that the T3D S1 gene, which encodes viral attachment protein σ 1, is associated with larger apoptosis induction in L929 cells (Tyler et al., 1995), a study by Sherry and colleagues that showed that viral core proteins λ2, μ2, and σ2 were associated with IFN-β induction in cardiac myocyte cultures (Sherry et al., 1998), a study by Clarke and colleagues that revealed that the T3D S1 and M2 genes (encoding σ1 and μ1, respectively) were associated with apoptosis and JNK activation (Clarke et al., 2001), a study by Miller and colleagues that demonstrated that protein ubiquitination was more greatly enhanced in CV-1 cells infected with MRV clones that contained primarily the T3D μ2 protein, with some contribution by the λ 2 and λ 3 proteins (Miller et al., 2004), and a study by Zurney and colleagues that demonstrated the T1L μ2 protein represses IFN-mediated induction of various interferon-stimulated genes in L929 cells and leads to IRF9 accumulation in the nucleus (Zurney et al., 2009). The present study contributes a large number of cellular proteins that are differentially regulated by T1L and T3D. Seventeen host proteins (11 in the cytosol and six in the nucleus) are up-regulated ≥1.5-fold by T1L infection but not by T3D infection. Most of these uniquely T1L-induced up-regulated proteins (1433E, 2AAA, BAF, CAPR1, CH60, DHAK, EF2, ERH, H2B1M, MATR3, SCOC, SYIC, and U2AF2, and the ribosomal proteins RM10, RM44, and RS7) are involved in binding various molecules including ATP, DNA, RNA and/or protein. BAF, CH60, and 1433E have previously been implicated in virus-host interactions. Two proteins (BAF and H2B1M) are involved in chromatin structure/organization and one protein (H2B1M) is also involved in DNA damage/repair. Sixteen host proteins (seven in the cytosol and nine in the nucleus) are down-regulated ≥1.5-fold (to ≤0.667) by T1L infection but not by T3D infection. These include five proteins (H2AY, H2B2F, HMGN1, HMGN2, and PARP1) involved in chromatin structure/organization and four proteins (H2B2F, HMGN1, PARP1, XRCC5) involved in DNA damage/repair. Many T1L-induced down-regulated proteins (DHX15, HMGN2, PARP1, PRP6, RBM3, SRSF2, and XRCC5) are involved in RNA binding/processing/splicing. Twenty four host proteins (17 in the cytosol and seven in the nucleus) are up-regulated ≥1.5-fold by T3D infection but not by T1L infection, and four of these proteins (Mx1, ISG15, IFIT1, and STAT1) are up-regulated >3.5-fold by T3D. The T3D-induced highly up-regulated proteins (Mx1, ISG15, IFIT1, and STAT1) and two other proteins (IFIT3 and DDX58) are involved in interferon signaling. Numerous T3D-induced up-regulated proteins are also associated with apoptosis (ACTN4, IFIT3, Mx1, and STAT1) and either anti-viral responses or virus-host interactions (AP2A1, CH60, DDX58, ELOA1, IFIT1, IFIT3, ISG15, Mx1, RBP2, and STAT1). No T3D-induced up-regulated proteins are associated with chromatin structure/organization or RNA processing/splicing, although five T3D-induced down-regulated proteins (DEK, H2B1N, H4, HNRPR, and RCC1) are associated with those functions. In addition, of the 10 proteins (four in the cytosol and six in the nucleus) that are down-regulated ≥1.5-fold by T3D infection but not by T1L infection, DEK, RCC1, and RS18 have been associated with viral processes.

Viruses are notable for inducing a variety of alterations in their host cells, ranging from asymptomatic in some cases to highly pathogenic in others. Different strains of the same virus can also induce differences in pathology. Although the reovirus T1L strain often produces higher titers at initial sites of infection than the T3D strain (Farone et al., 1996), the strains travel by different routes to the murine brain and induce different diseases, with T3D usually leading to more lethal infection (Tyler et al., 1986; Tyler, 1998). These observations correlate with our observation that T3D induced more host protein dysregulation than T1L, particularly in immune regulatory molecules (Table 1). These cellular protein alterations may also play a role in oncolytic potential of reovirus. Enhanced oncolytic potential is based upon cellular activation, including *ras* activation (Strong et al., 1998), p53 stabilization (Pan et al., 2011), and cathepsin activity levels (Terasawa et al., 2015), some of which play roles in apoptosis (Pan et al., 2011) and autophagy (Thirukkumaran et al., 2013), processes found to be altered in this study.

In conclusion, comparative iTRAQ analyses demonstrate that reoviruses T1L and T3D induce different proteomic responses in infected HeLa cells, with the T3D clone inducing higher dysregulation of various cellular signaling pathways.

## Author contributions

PE and KC designed experiments, and all authors performed experimental work and edited the manuscript.

### Conflict of interest statement

The authors declare that the research was conducted in the absence of any commercial or financial relationships that could be construed as a potential conflict of interest.

## References

[B1] Aggarwal K. Choe L. H. Lee K. H. (2006). Shotgun proteomics using the iTRAQ isobaric tags. Brief Funct. Genomic. Proteomic. 5, 112–120. 10.1093/bfgp/ell018 16772272

[B2] Attoui H. Biagini P. Stirling J. Mertens P. P. C. Cantaloube J. F. Meyer A.. (2001). Sequence characterization of Ndelle virus genome segments 1, 5, 7, 8, and 10: evidence for reassignment to the genus Orthoreovirus, family Reoviridae. Biochem. Biophys. Res. Commun. 287, 583–588. 10.1006/bbrc.2001.5612 11554769

[B3] Baas T. Baskin C. R. Diamond D. L. Garcia-Sastre A. Bielefeldt-Ohmann H. Tumpey T. M.. (2006). Integrated molecular signature of disease: analysis of influenza virus-infected macaques through functional genomics and proteomics. J. Virol. 80, 10813–10828. 10.1128/JVI.00851-06 16928763 PMC1641753

[B4] Berard A. Coombs K. M. (2009). Mammalian reoviruses: propagation, quantification, and storage. Curr. Protoc. Microbiol. 2, 15C.1.1–15C.1.18. 10.1002/9780471729259.mc15c01s14 19653214

[B5] Berard A. R. Cortens J. P. Krokhin O. Wilkins J. A. Severini A. Coombs K. M. (2012). Characterization of the host response proteome after mammalian T1L reovirus infection. PLoS ONE 7:e51939. 10.1371/journal.pone.0051939 23240068 PMC3519901

[B7] Booy A. T. Haddow J. D. Ohlund L. B. Hardie D. B. Olafson R. W. (2005). Application of isotope coded affinity tag (ICAT) analysis for the identification of differentially expressed proteins following infection of Atlantic salmon (*Salmo salar*) with infectious hematopoietic necrosis virus (IHNV) or *Renibacterium salmoninarum* (BKD). J. Proteome Res. 4, 325–334 10.1021/pr049840t 15822907

[B8] Burgener A. Boutilier J. Wachihi C. Kimani J. Carpenter M. Westmacott G.. (2008). Identification of differentially expressed proteins in the cervical mucosa of HIV-1-resistant sex workers. J. Proteome Res. 7, 4446–4454. 10.1021/pr800406r 18707157

[B9] Choe L. H. Aggarwal K. Franck Z. Lee K. H. (2005). A comparison of the consistency of proteome quantitation using two-dimensional electrophoresis and shotgun isobaric tagging in *Escherichia coli* cells. Electrophoresis 26, 2437–2449. 10.1002/elps.200410336 15924362

[B10] Clarke P. Leser J. S. Bowen R. A. Tyler K. L. (2014). Virus-induced transcriptional changes in the brain include the differential expression of genes associated with interferon, apoptosis, interleukin 17 receptor A, and glutamate signaling as well as Flavivirus-specific upregulation of tRNA synthetases. Mbio 5:e00902-14. 10.1128/mBio.00902-14 24618253 PMC3952157

[B11] Clarke P. Meintzer S. M. Widmann C. Johnson G. L. Tyler K. L. (2001). Reovirus infection activates JNK and the JNK-dependent transcription factor c-Jun. J. Virol. 75, 11275–11283. 10.1128/JVI.75.23.11275-11283.2001 11689607 PMC114712

[B12] Coffey M. C. Strong J. E. Forsyth P. A. Lee P. W. (1998). Reovirus therapy of tumors with activated Ras pathway. Science 282, 1332–1334. 10.1126/science.282.5392.1332 9812900

[B13] Coombs K. M. (2011a). Quantitative proteomics of complex mixtures. Expert Rev. Proteomics 8, 659–677. 10.1586/epr.11.55 21999835

[B14] Coombs K. M. (2011b). Reoviruses, in Encyclopedia of Life Sciences, eds Mahy B. W. J. van Regenmortel M. H. V. (London: Macmillan), 390–399.

[B15] Coombs K. M. (2013). HeLa cell response proteome alterations induced by mammalian reovirus T3D infection. Virol. J. 10:202. 10.1186/1743-422X-10-202 23799967 PMC3847587

[B16] Coombs K. M. Berard A. Xu W. Krokhin O. Meng X. Cortens J. P.. (2010). Quantitative proteomic analyses of influenza virus-infected cultured human lung cells. J. Virol. 84, 10888–10906. 10.1128/JVI.00431-10 20702633 PMC2950599

[B17] Danthi P. Guglielmi K. M. Kirchner E. Mainou B. Stehle T. Dermody T. S. (2010). From touchdown to transcription: the reovirus cell entry pathway. Curr. Top. Microbiol. Immunol. 343, 91–119. 10.1007/82_2010_32 20397070 PMC4714703

[B18] Debiasi R. L. Clarke P. Meintzer S. Jotte R. Kleinschmidt-Demasters B. K. Johnson G. L.. (2003). Reovirus-induced alteration in expression of apoptosis and DNA repair genes with potential roles in viral pathogenesis. J. Virol. 77, 8934–8947. 10.1128/JVI.77.16.8934-8947.2003 12885910 PMC167209

[B19] de Hoog C. L. Foster L. J. Mann M. (2004). RNA and RNA binding proteins participate in early stages of cell spreading through spreading initiation centers. Cell 117, 649–662. 10.1016/S0092-8674(04)00456-8 15163412

[B20] Dennis G. Sherman B. T. Hosack D. A. Yang J. Gao W. Lane H. C.. (2003). DAVID: database for annotation, visualization, and integrated discovery. Genome Biol. 4:P3. 10.1186/gb-2003-4-5-p3 12734009 PMC193660

[B21] Dermody T. S. Parker J. S. L. Sherry B. (2013). Orthoreoviruses, in Fields Virology, 6th Edn., eds Knipe D. Howley P. M. (Philadelphia, PA: Lippincott Williams and Wilkins), 1304–1346.

[B22] Dwivedi R. C. Dhindsa N. Krokhin O. V. Cortens J. Wilkins J. A. El Gabalawy H. S. (2009). The effects of infliximab therapy on the serum proteome of rheumatoid arthritis patients. Arthritis Res. Ther. 11:R32. 10.1186/ar2637 19265537 PMC2688177

[B23] Dwivedi R. C. Spicer V. Harder M. Antonovici M. Ens W. Standing K. G.. (2008). Practical implementation of 2D HPLC scheme with accurate peptide retention prediction in both dimensions for high-throughput bottom-up proteomics. Anal. Chem. 80, 7036–7042. 10.1021/ac800984n 18686972

[B24] Estes M. K. Kapikian A. Z. (2007). Rotaviruses, in Fields Virology, eds Knipe D. M. Howley P. M. (Philadelphia, PA: Lippincott Williams and Wilkins), 1917–1974.

[B25] Everley P. A. Krijgsveld J. Zetter B. R. Gygi S. P. (2004). Quantitative cancer proteomics: stable isotope labeling with amino acids in cell culture (SILAC) as a tool for prostate cancer research. Mol. Cell Proteomics 3, 729–735. 10.1074/mcp.M400021-MCP200 15102926

[B26] Farone A. L. Frevert C. W. Farone M. B. Morin M. J. Fields B. N. Paulauskis J. D.. (1996). Serotype-dependent induction of pulmonary neutrophilia and inflammatory cytokine gene expression by reovirus. J. Virol. 70, 7079–7084. 8794353 10.1128/jvi.70.10.7079-7084.1996 PMC190759

[B27] Forsyth P. Roldan G. George D. Wallace C. Palmer C. A. Morris D.. (2008). A phase I trial of intratumoral administration of reovirus in patients with histologically confirmed recurrent malignant gliomas. Mol. Ther. 16, 627–632. 10.1038/sj.mt.6300403 18253152

[B28] Geiss G. K. Salvatore M. Tumpey T. M. Carter V. S. Wang X. Y. Basler C. F.. (2002). Cellular transcriptional profiling in influenza A virus-infected lung epithelial cells: the role of the nonstructural NS1 protein in the evasion of the host innate defense and its potential contribution to pandemic influenza. Proc. Natl. Acad. Sci. U.S.A. 99, 10736–10741. 10.1073/pnas.112338099 12149435 PMC125029

[B29] Gilar M. Olivova P. Daly A. E. Gebler J. C. (2005). Two-dimensional separation of peptides using RP-RP-HPLC system with different pH in first and second separation dimensions. J. Sep. Sci. 28, 1694–1703. 10.1002/jssc.200500116 16224963

[B30] Huang D. W. Sherman B. T. Lempicki R. A. (2009). Systematic and integrative analysis of large gene lists using DAVID bioinformatics resources. Nat. Protoc. 4, 44–57. 10.1038/nprot.2008.211 19131956

[B31] Jiang J. Opanubi K. J. Coombs K. M. (2012). Non-biased enrichment does not improve quantitative proteomic delineation of reovirus T3D-infected HeLa cell protein alterations. Front. Microbiol. 3:310. 10.3389/fmicb.2012.00310 23024642 PMC3447384

[B32] Keshamouni V. G. Jagtap P. Michailidis G. Strahler J. R. Kuick R. Reka A. K.. (2009). Temporal quantitative proteomics by iTRAQ 2D-LC-MS/MS and corresponding mRNA expression analysis identify post-transcriptional modulation of actin-cytoskeleton regulators during TGF-beta-Induced epithelial-mesenchymal transition. J. Proteome Res. 8, 35–47. 10.1021/pr8006478 19118450

[B33] Kobasa D. Jones S. M. Shinya K. Kash J. C. Copps J. Ebihara H.. (2007). Aberrant innate immune response in lethal infection of macaques with the 1918 influenza virus. Nature 445, 319–323. 10.1038/nature05495 17230189

[B34] Kroeker A. L. Ezzati P. Halayko A. J. Coombs K. M. (2012). Response of primary human airway epithelial cells to influenza infection—a quantitative proteomic study. J. Proteome Res. 11, 4132–4136. 10.1021/pr300239r 22694362 PMC3411195

[B35] Li L. Sevinsky J. R. Rowland M. D. Bundy J. L. Stephenson J. L. Sherry B. (2010). Proteomic analysis reveals virus-specific Hsp25 modulation in cardiac myocytes. J. Proteome Res. 9, 2460–2471. 10.1021/pr901151k 20196617 PMC2866012

[B36] Lucitt M. B. Price T. S. Pizarro A. Wu W. Yocum A. K. Seiler C.. (2008). Analysis of the zebrafish proteome during embryonic development. Mol. Cell. Proteomics 7, 981–994. 10.1074/mcp.M700382-MCP200 18212345 PMC2401336

[B37] Mendez I. I. Hermann L. L. Hazelton P. R. Coombs K. M. (2000). A comparative analysis of freon substitutes in the purification of reovirus and calicivirus. J. Virol. Methods 90, 59–67. 10.1016/S0166-0934(00)00217-2 11011081

[B38] Mertens P. P. C. Attoui H. Duncan R. Dermody T. S. (2005). Reoviridae, in Virus Taxonomy. Eighth Report of the International Committee on Taxonomy of Viruses, eds Fauquet C. M. Mayo M. A. Maniloff J. Desselberger U. Ball L. A. (London: Elsevier/Academic Press), 447–454.

[B39] Miller C. L. Parker J. S. Dinoso J. B. Piggott C. D. Perron M. J. Nibert M. L. (2004). Increased ubiquitination and other covariant phenotypes attributed to a strain- and temperature-dependent defect of reovirus core protein mu2. J. Virol. 78, 10291–10302. 10.1128/JVI.78.19.10291-10302.2004 15367595 PMC516405

[B40] O'donnell S. M. Holm G. H. Pierce J. M. Tian B. Watson M. J. Chari R. S.. (2006). Identification of an NF-kappaB-dependent gene network in cells infected by mammalian reovirus. J. Virol. 80, 1077–1086. 10.1128/JVI.80.3.1077-1086.2006 16414985 PMC1346919

[B41] Ong S. E. Blagoev B. Kratchmarova I. Kristensen D. B. Steen H. Pandey A.. (2002). Stable isotope labeling by amino acids in cell culture, SILAC, as a simple and accurate approach to expression proteomics. Mol. Cell. Proteomics 1, 376–386. 10.1074/mcp.M200025-MCP200 12118079

[B42] Ong S. E. Mann M. (2005). Mass spectrometry-based proteomics turns quantitative. Nat. Chem. Biol. 1, 252–262. 10.1038/nchembio736 16408053

[B43] Pan D. Pan L. Z. Hill R. Marcato P. Shmulevitz M. Vassilev L. T.. (2011). Stabilisation of p53 enhances reovirus-induced apoptosis and virus spread through p53-dependent NF-kappa B activation. Br. J. Cancer 105, 1012–1022. 10.1038/bjc.2011.325 21863032 PMC3185941

[B44] Poggioli G. J. Debiasi R. L. Bickel R. Jotte R. Spalding A. Johnson G. L.. (2002). Reovirus-induced alterations in gene expression related to cell cycle regulation. J. Virol. 76, 2585–2594. 10.1128/JVI.76.6.2585-2594.2002 11861824 PMC135961

[B45] Pradet-Balade B. Boulme F. Beug H. Mullner E. W. Garcia-Sanz J. A. (2001). Translation control: bridging the gap between genomics and proteomics? Trends Biochem. Sci. 26, 225–229. 10.1016/S0968-0004(00)01776-X 11295554

[B46] Prange A. Proefrock D. (2008). Chemical labels and natural element tags for the quantitative analysis of bio-molecules. J. Anal. Atomic Spectrom. 23, 432–459 10.1039/b717916m

[B47] Roy P. (2007). Orbiviruses, in Fields Virology, eds Knipe D. M. Howley P. M. (Philadelphia, PA: Lippencott Williams and Wilkins), 1975–1997.

[B48] Sherry B. Torres J. Blum M. A. (1998). Reovirus induction of and sensitivity to beta interferon in cardiac myocyte cultures correlate with induction of myocarditis and are determined by viral core proteins. J. Virol. 72, 1314–1323. 9445032 10.1128/jvi.72.2.1314-1323.1998 PMC124610

[B49] Smith R. E. Zweerink H. J. Joklik W. K. (1969). Polypeptide components of virions, top component and cores of reovirus type 3. Virology 39, 791–810. 10.1016/0042-6822(69)90017-8 4311639

[B50] Stewart J. J. White J. T. Yan X. Collins S. Drescher C. W. Urban N. D.. (2006). Proteins associated with Cisplatin resistance in ovarian cancer cells identified by quantitative proteomic technology and integrated with mRNA expression levels. Mol. Cell. Proteomics 5, 433–443. 10.1074/mcp.M500140-MCP200 16319398

[B51] Strong J. E. Coffey M. C. Tang D. Sabinin P. Lee P. W. (1998). The molecular basis of viral oncolysis: usurpation of the Ras signaling pathway by reovirus. EMBO J. 17, 3351–3362. 10.1093/emboj/17.12.3351 9628872 PMC1170673

[B52] Summers W. A. Wilkins J. A. Dwivedi R. C. Ezzati P. Court D. A. (2012). Mitochondrial dysfunction resulting from the absence of mitochondrial porin in *Neurospora crassa*. Mitochondrion 12, 220–229. 10.1016/j.mito.2011.09.002 21946565

[B53] Szklarczyk D. Franceschini A. Kuhn M. Simonovic M. Roth A. Minguez P.. (2011). The STRING database in 2011: functional interaction networks of proteins, globally integrated and scored. Nucleic Acids Res. 39, D561–D568. 10.1093/nar/gkq973 21045058 PMC3013807

[B54] Terasawa Y. Hotani T. Katayama Y. Tachibana M. Mizuguchi H. Sakurai F. (2015). Activity levels of cathepsins B and L in tumor cells are a biomarker for efficacy of reovirus-mediated tumor cell killing. Cancer Gene Ther. [Epub ahead of print]. 10.1038/cgt.2015.4 25633482

[B55] Thirukkumaran C. M. Nodwell M. J. Hirasawa K. Shi Z. Q. Diaz R. Luider J.. (2010). Oncolytic viral therapy for prostate cancer: efficacy of reovirus as a biological therapeutic. Cancer Res. 70, 2435–2444. 10.1158/0008-5472.CAN-09-2408 20215509

[B56] Thirukkumaran C. M. Shi Z. Q. Luider J. Kopciuk K. Gao H. Bahlis N.. (2013). Reovirus modulates autophagy during oncolysis of multiple myeloma. Autophagy 9, 413–414. 10.4161/auto.22867 23322106 PMC3590261

[B57] Tian Q. Stepaniants S. B. Mao M. Weng L. Feetham M. C. Doyle M. J.. (2004). Integrated genomic and proteomic analyses of gene expression in mammalian cells. Mol. Cell. Proteomics 3, 960–969. 10.1074/mcp.M400055-MCP200 15238602

[B58] Tyler K. L. (1998). Pathogenesis of reovirus infections of the central nervous system. Curr. Top. Microbiol. Immunol. 233, 93–124. 10.1007/978-3-642-72095-6_6 9599934

[B59] Tyler K. L. Leser J. S. Phang T. L. Clarke P. (2010). Gene expression in the brain during reovirus encephalitis. J. Neurovirol. 16, 56–71. 10.3109/13550280903586394 20158406 PMC2891017

[B60] Tyler K. L. McPhee D. A. Fields B. N. (1986). Distinct pathways of viral spread in the host determined by reovirus S1 gene segment. Science 233, 770–774. 10.1126/science.3016895 3016895

[B61] Tyler K. L. Squier M. K. Rodgers S. E. Schneider B. E. Oberhaus S. M. Grdina T. A.. (1995). Differences in the capacity of reovirus strains to induce apoptosis are determined by the viral attachment protein sigma 1. J. Virol. 69, 6972–6979. 7474116 10.1128/jvi.69.11.6972-6979.1995 PMC189616

[B62] Von Mering C. Jensen L. J. Kuhn M. Chaffron S. Doerks T. Kruger B.. (2007). STRING 7—recent developments in the integration and prediction of protein interactions. Nucleic Acids Res. 35, D358–D362. 10.1093/nar/gkl825 17098935 PMC1669762

[B63] Yan W. Lee H. Yi E. C. Reiss D. Shannon P. Kwieciszewski B. K.. (2004). System-based proteomic analysis of the interferon response in human liver cells. Genome Biol. 5:R54. 10.1186/gb-2004-5-8-r54 15287976 PMC507879

[B64] Yates J. R. Ruse C. I. Nakorchevsky A. (2009). Proteomics by mass spectrometry: approaches, advances, and applications. Ann. Rev. Biomed. Eng. 11, 49–79. 10.1146/annurev-bioeng-061008-124934 19400705

[B65] Zhang J. Niu D. Sui J. Ching C. B. Chen W. N. (2009). Protein profile in hepatitis B virus replicating rat primary hepatocytes and HepG2 cells by iTRAQ-coupled 2-D LC-MS/MS analysis: insights on liver angiogenesis. Proteomics 9, 2836–2845. 10.1002/pmic.200800911 19405029

[B66] Zieske L. R. (2006). A perspective on the use of iTRAQ reagent technology for protein complex and profiling studies. J. Exp. Bot. 57, 1501–1508. 10.1093/jxb/erj168 16574745

[B67] Zurney J. Kobayashi T. Holm G. H. Dermody T. S. Sherry B. (2009). Reovirus mu2 protein inhibits interferon signaling through a novel mechanism involving nuclear accumulation of interferon regulatory factor 9. J. Virol. 83, 2178–2187. 10.1128/JVI.01787-08 19109390 PMC2643726

