# Supplementary material for: Comparative proteomic analyses demonstrate enhanced interferon and STAT-1 activation in reovirus T3D-infected HeLa cells
Source: Front Cell Infect Microbiol. 2015 Apr 7;5:30. doi: 10.3389/fcimb.2015.00030 (PMC4388007; doi:10.3389/fcimb.2015.00030)
Supplement: Supplementary file 1 [file Image1.PDF]

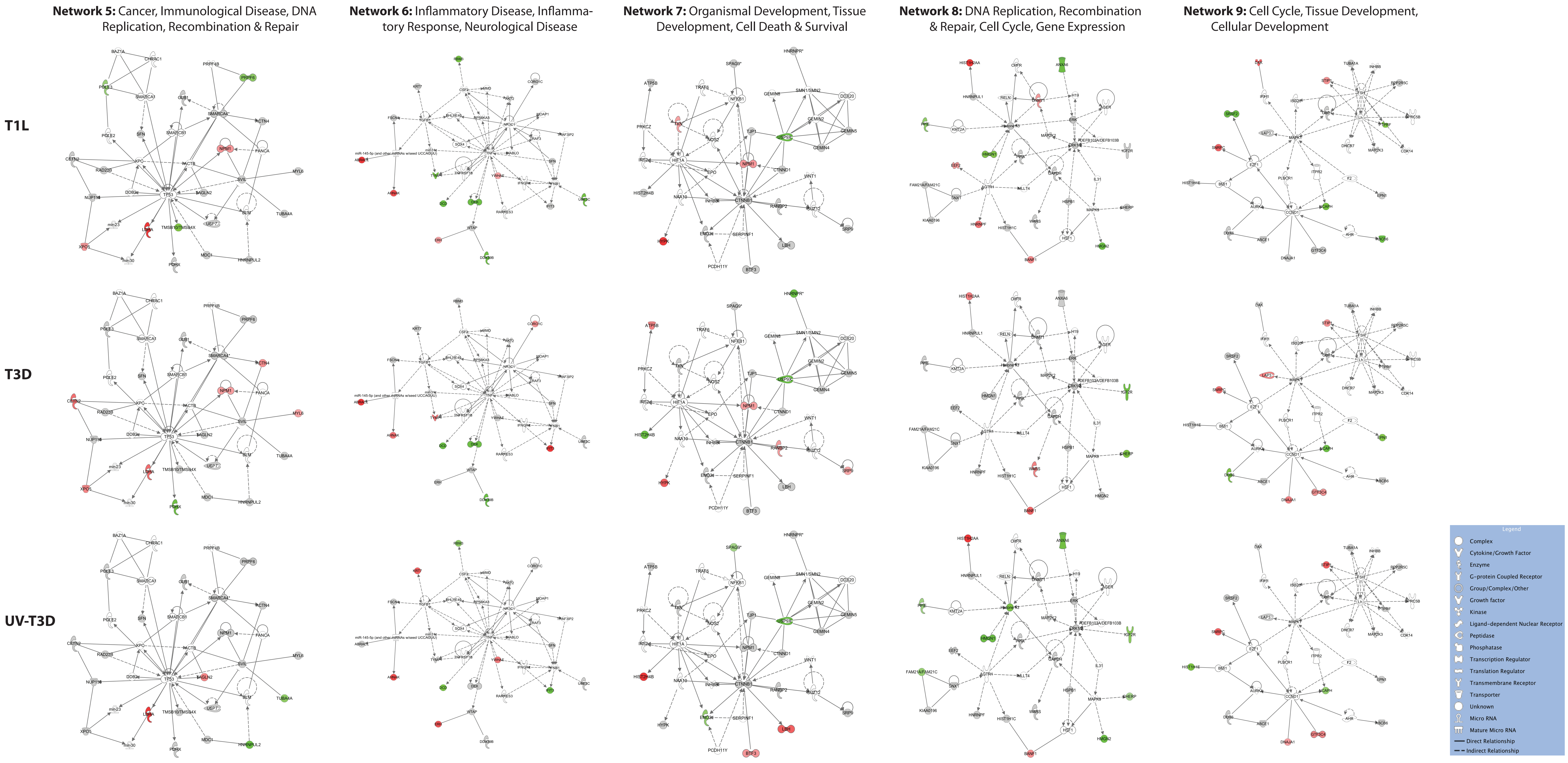

Network 5: Cancer, Immunological Disease, DNA Replication, Recombination & Repair
Network 6: Inflammatory Disease, Inflammatory Response, Neurological Disease
Network 7: Organismal Development, Tissue Development, Cell Death & Survival
Network 8: DNA Replication, Recombination & Repair, Cell Cycle, Gene Expression
Network 9: Cell Cycle, Tissue Development, Cellular Development
T1L
T3D
UV-T3D
Legend
Complex
Cytokine/Growth Factor
Enzyme
G-protein Coupled Receptor
Group/Complex/Other
Growth factor
Kinase
Ligand-dependent Nuclear Receptor
Peptidase
Phosphatase
Transcription Regulator
Translation Regulator
Transmembrane Receptor
Transporter
Unknown
Micro RNA
Mature Micro RNA
Direct Relationship
Indirect Relationship
