# Supplementary material for: Comparative proteomic analyses demonstrate enhanced interferon and STAT-1 activation in reovirus T3D-infected HeLa cells
Source: Front Cell Infect Microbiol. 2015 Apr 7;5:30. doi: 10.3389/fcimb.2015.00030 (PMC4388007; doi:10.3389/fcimb.2015.00030)

Supplementary Figure S2. Additional differentially regulated canonical pathways

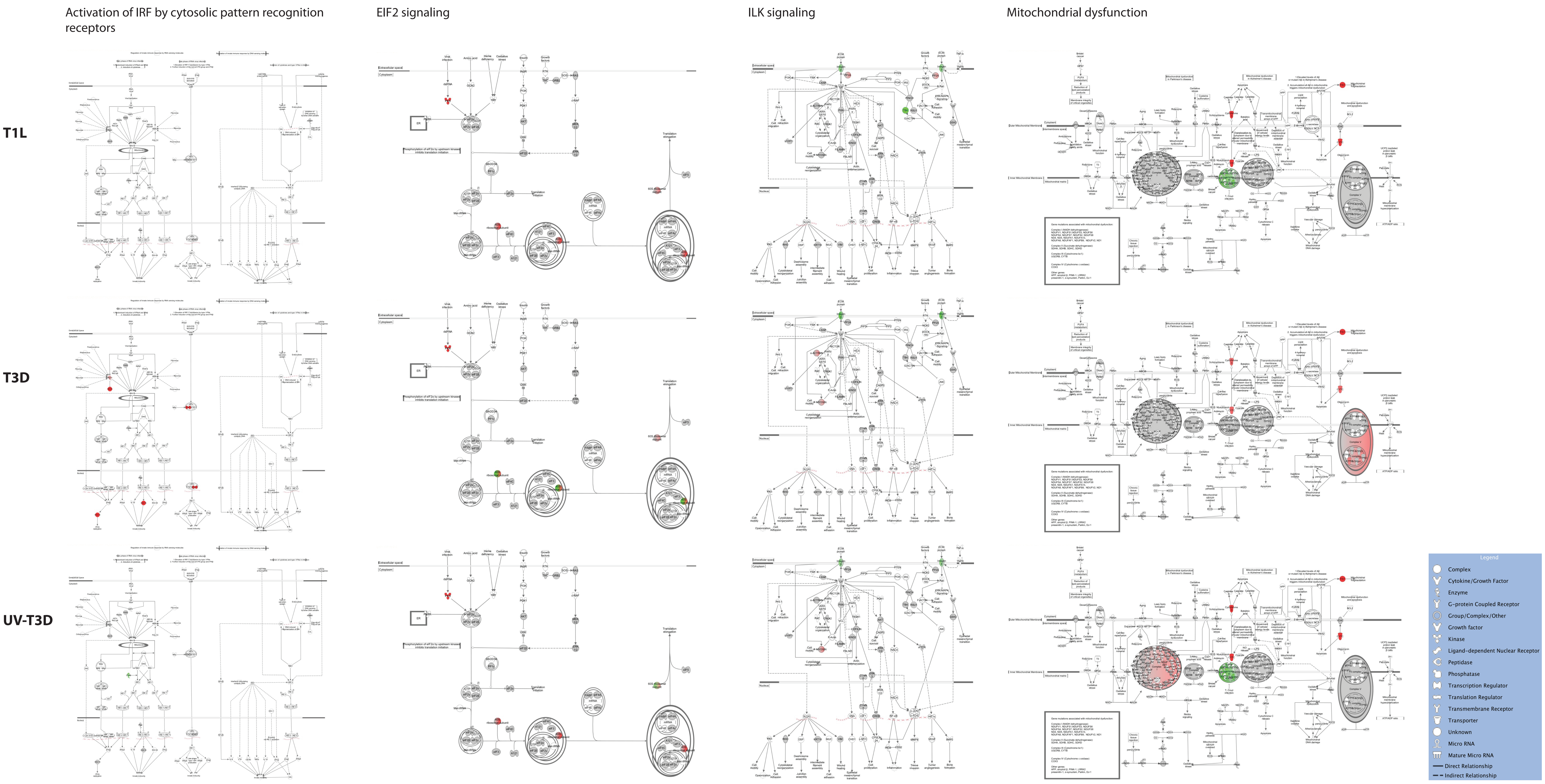

Supplement: Supplementary file 2 [file Image2.PDF]
